# A Teleost CXCL10 Is Both an Immunoregulator and an Antimicrobial

**DOI:** 10.3389/fimmu.2022.917697

**Published:** 2022-06-20

**Authors:** Huili Li, Yuanyuan Sun, Li Sun

**Affiliations:** ^1^ Chinese Academy of Sciences (CAS) and Shandong Province Key Laboratory of Experimental Marine Biology, Institute of Oceanology; CAS Center for Ocean Mega-Science, Chinese Academy of Sciences, Qingdao, China; ^2^ Laboratory for Marine Biology and Biotechnology, Pilot National Laboratory for Marine Science and Technology, Qingdao, China; ^3^ College of Earth and Planetary Sciences, University of Chinese Academy of Sciences, Beijing, China

**Keywords:** *Paralichthys olivaceus*, chemokine, bacterial pathogen, antimicrobial, immune defense

## Abstract

Chemokines are a group of cytokines that play important roles in cell migration, inflammation, and immune defense. In this study, we identified a CXC chemokine, CXCL10, from Japanese flounder *Paralichthys olivaceus* (named PoCXCL10) and investigated its immune function. Structurally, PoCXCL10 possesses an N-terminal coil, three β-strands, and a C-terminal α-helix with cationic and amphipathic properties. PoCXCL10 expression occurred in multiple tissues and was upregulated by bacterial pathogens. Recombinant PoCXCL10 (rPoCXCL10) promoted the migration, cytokine expression, and phagocytosis of flounder peripheral blood leukocytes (PBLs). rPoCXCL10 bound to and inhibited the growth of a variety of common Gram-negative and Gram-positive fish pathogens. rPoCXCL10 killed the pathogens by causing bacterial membrane permeabilization and structure destruction. When introduced *in vivo*, rPoCXCL10 significantly inhibited bacterial dissemination in fish tissues. A peptide derived from the C-terminal α-helix exhibited bactericidal activity and competed with rPoCXCL10 for bacterial binding. Deletion of the α-helix affected the *in vitro* bactericidal activity but not the chemotaxis or *in vivo* antimicrobial activity of PoCXCL10. Together, these results indicate that PoCXCL10 exerts the role of both an immunoregulator and a bactericide/bacteriostatic *via* different structural domains. These findings provide new insights into the immune function and working mechanism of fish CXC chemokines.

## Introduction

Chemokines are a group of small (8 to 12 kDa) proteins with the ability to induce cell migration and immune responses by binding to G protein-coupled receptors on the target cells (1–5). Chemokines share a common structure, which is stabilized by the disulfide bonds between conserved cysteine residues (6). In mammals, on the basis of the number and distinctive pattern of the N-terminal cysteine residues, chemokines are categorized into four subfamilies: CXC, CC, C (also called XC), and CX_3_C (7). In fish, the CXC, CC, and C subfamilies have been identified, but no CX_3_C has been reported (8, 9). The CC and CXC chemokines are the two major subfamilies in both mammals and fish (9).

The functions of many chemokines have been reported. “Homeostatic” chemokines, such as CCL16 and CXCL13, are constitutively expressed and play an important role in regulating the homing and maturation of immune cells in homeostasis (10, 11). The CXCL12-CXCR4 axis is known to be vital to the development of the central nervous system in zebrafish and for the development of coronary artery in mice (12–14). “Inducible” chemokines, such as CCL2 and CXCL8, are induced by inflammatory signals or pathogen infection, and act as key mediators in innate and adaptive immunity (15, 16). Some mammalian chemokines have been shown to possess direct bactericidal activities (17). In fish, the functions of a number of chemokines have been studied. For example, turbot *Scophthalmus maximus* CCL19 was found to exhibit chemotactic activity and immunoregulatory property, which enhanced host resistance against viral and bacterial infection (18); tongue sole *Cynoglossus semilaevis* CCL21 was able to recruit leukocytes and involved in antibacterial defense (19); rainbow trout *Oncorhynchus mykiss* CK12a contributed to host antiviral immune response (20). To date, bactericidal activity has been observed only in rainbow trout CK11 and grass carp *Ctenopharyngodon Idella* CXCL20a/b (21–23).

CXCL10 is a member of the interferon-inducible CXC chemokine subfamily. In mammals, CXCL10 induces the migration of various immune cells, including T cells, natural killer cells, macrophages, and dendritic cells (24–27), and enhances T_H_1 cell generation during colitis development (28). In steatohepatitis, CXCL10 contributes to hepatic apoptosis and employs transcription factor nuclear factor kappa-B (NF-κB) to mediate inflammation (29). Recently, CXCL10 was reported to play a critical role in mediating the production of the proinflammatory cytokines IL-12 and IL-23 (30). In addition, human CXCL10 is known to possess bactericidal activity against *Escherichia coli*, *Listeria monocytogenes*, *Bacillus anthracis* and *Staphylococcus aureus* (31–34). In teleost, CXCL10 has been identified in rainbow trout *Oncorhynchus mykiss*, channel catfish *Ictalurus punctatus*, turbot *Scophthalmus maximus*, red sea bream *Pagrus major*, and Nile tilapia *Oreochromis niloticus* (35–39). However, the biological function of teleost CXCL10 at the protein level remains poorly understood.

Japanese flounder *Paralichthys olivaceus* is one of the major cultured marine fish species in Asia. In this study, we identified a CXCL10 homologue from flounder and examined its chemotactic activity and antimicrobial activity in relationship to its structure. In addition, we also examined the bactericidal effect of a CXCL10-derived peptide. Our results indicate an important role of CXCL10 in host immune defense against bacterial infection and expand the understanding of the biological function of teleost chemokines.

## Materials and Methods

### Animals

Clinically healthy Japanese flounder *Paralichthys olivaceus* (average weight of 20 g) were purchased from a local commercial fish farm in Shandong Province, China. The fish were acclimatized at 19-20°C in aerated seawater for at least one week before the experiments. Before tissue collection, the fish were euthanized with tricaine methanesulfonate (Sigma, St. Louis, MO, USA) as reported previously (40).

### Sequence Analysis of PoCXCL10

The amino acid sequence of PoCXCL10 (accession no. XP_019935349.1) was analyzed with the basic local alignment search tool (BLAST) program at the National Center for Biotechnology Information (NCBI). The signal peptide was predicted with the SignalP5.0 program (http://www.cbs.dtu.dk/services/SignalP/). The molecular mass (Mw) and theoretical isoelectric point (pI) were obtained using the ExPASy molecular biology server (https://web.expasy.org/compute_pi/). Multiple sequence alignment was created with DNAMAN (version 7.0.2.176, Lynnon Biosoft, San Ramon, CA, USA) software. Phylogenetic analysis was constructed using the Neighbor-joining algorithm of MEGA 6.0 software (Tempe, AZ, USA).

### Protein Structure Prediction and Analysis

The three-dimensional (3D) model of PoCXCL10 was predicted with Iterative Threading Assembly Refinement (https://zhanglab.ccmb.med.umich.edu/I-TASSER/) (41). The 3D models were visualized with PyMOL (PyMOL Molecular Graphics System, Version 2.5.0, Schrödinger). Electrostatic surface potentials and solvent accessibility representation were calculated with Adaptive Poisson-Boltzmann Solver (APBS) software and displayed with Visual Molecular Dynamics (VMD) (42, 43). Helical wheel projection of the peptide was performed with HeliQuest (https://heliquest.ipmc.cnrs.fr/cgi-bin/ComputParams.py).

### Determination of PoCXCL10 Expression by Quantitative Real-Time Reverse Transcription-Polymerase Chain Reaction (qRT-PCR)

qRT-PCR was performed to determine the expression of PoCXCL10 in fish tissues under normal physiological conditions as reported previously (44). Briefly, tissue samples were isolated from the gill, blood, muscle, head kidney, intestine, brain, heart, spleen and liver of healthy Japanese flounder. Total RNA was extracted using TRIzol Reagent (Invitrogen, Carlsbad, CA, USA) and used for cDNA synthesis with the First Strand cDNA Synthesis Kit (ToYoBo, Osaka, Japan) according to the manufacturer’s protocol. qRT-PCR was carried out with Eppendorf Mastercycler^®^ ep gradient S (Eppendorf, Hamburg, Germany) using ChamQ Universal SYBR qPCR master mix (Vazyme, Najing, China). Gene expression was calculated using comparative threshold cycle method (2^−ΔΔCT^) and normalized to that of β-actin.

To examine the expression of PoCXCL10 during bacterial infection, *Edwardsiella tarda*, *Vibrio anguillarum* and *Vibrio harveyi* were cultured in Luria-Bertani (LB) medium at 28°C to logarithmic phase, and resuspended in phosphate-buffered saline (PBS) to 1×10^7^ CFU/ml. Japanese flounder were infected with 100 µL *E. tarda*, *V. anguillarum*, *V. harveyi* or PBS (control). At 6, 12, 24, and 48 h post infection (hpi), spleen, liver and head kidney were collected. qRT-PCR analysis of PoCXCL10 expression in the collected tissues was performed as described above. 18S ribosomal RNA (18S rRNA), α-tubulin, and glyceraldehyde-3-phosphate dehydrogenase (GAPDH) were used as internal references in head kidney, spleen, and liver, respectively (45). The sequences of the primers used for qRT-PCR are listed in **Table S1**. All qRT-PCR assays were performed in triplicate.

### Protein Expression and Purification

To obtain recombinant proteins of PoCXCL10 (rPoCXCL10) and a mutant PoCXCL10 lacking the C-terminal α-helix region (rPoCXCL10M), the coding sequence of PoCXCL10 and PoCXCL10M were amplified by PCR with the primer pairs PoCXCL10-F/PoCXCL10-R and PoCXCL10M-F/PoCXCL10M-R, respectively (**Table S1**). The PCR products were inserted into pET28a-SUMO plasmid (46) at between the BamH I and Xho I sites. The recombinant plasmids were introduced into *E. coli* Transetta (DE3) (TransGen Biotech, Beijing, China) *via* transformation. Recombinant proteins were purified from the transformants as reported previously (44) with slight adjustments. Briefly, the transformants were cultured in LB medium at 37°C to an OD_600_ of 0.5. Isopropyl-β-D-thiogalactopyranoside was added to the medium to a final concentration of 0.3 mM. The sample was then transferred to 16°C and cultured for 16 h. Then the cells were collected by centrifugation (6000 rpm) for 10 min, and Sumo-tagged rPoCXCL10/rPoCXCL10M and recombinant Sumo tag (rSumo) (as a control protein) were purified using nickel-nitrilotriacetic acid (Ni-NTA) column (QIAGEN, Valencia, USA). The Sumo-tag was cut off with Sumo protease (ubiquitin-like proteases, ULP) as previously reported (46), resulting in tag-free rPoCXCL10 and rPoCXCL10M. The recombinant proteins were analyzed by sodium dodecyl sulfate-polyacrylamide gel electrophoresis (SDS-PAGE) and visualized after staining with Coomassie brilliant blue R-250 (**Figure S1**).

### Binding of Protein/Peptide to Bacteria and Bacterial Components

Protein-bacteria interaction was determined with enzyme-linked immunosorbent assay (ELISA) as reported previously (44) with slight adjustments. Briefly, bacteria (*Bacillus subtilis*, *Micrococcus luteus*, *Streptococcus iniae*, *V. harveyi*, *V. anguillarum*, *E. tarda*, and *Pseudomonas fluorescens*) were cultured to logarithmic phase and resuspended in coating buffer (15 mM Na_2_CO_3_, 35 mM NaHCO_3_, pH 9.6) to a final concentration of 1×10^8^ CFU/ml. The bacterial suspensions were added to 96-well plates (100 μl/well), and the plates were incubated at 4 °C for overnight. After incubation, the plates were blocked with 5% skim milk and incubated at room temperature for 2 h. The plates were washed three times with PBST (PBS containing 0.1% Tween-20). Then, 100 µL rPoCXCL10 at various concentrations (6.25 nM, 12.5 nM, 25 nM, 50 nM, and 100 nM) or rSumo (100 nM) or PBS (control) was added to the plates. The plates were incubated at room temperature for 2 h, followed by washing three times with PBST. Horseradish peroxidase (HRP)-conjugated mouse anti-Flag antibody (ABclonal, Wuhan, China) (2,000 dilution) was added to the plates, and the plates were incubated at 37 °C for 1 h. The plates were washed as above. Color development was performed with the 3, 3′, 5, 5′-tetramethylbenzidine (TMB) kit (Tiangen, Beijing, China), and absorbance at 450 nm was measured using a precision microplate reader (Molecular Devices, Toronto, Canada). The binding index was defined as follows: A_450_ of rPoCXCL10/A_450_ of PBS. To compare the bacteria-binding abilities of rPoCXCL10 and rPoCXCL10M, *V. anguillarum* was incubated with 100 nM rPoCXCL10, rPoCXCL10M, rSumo, or PBS for 2 h, and the bacteria-bound protein was determined by ELISA as above.

To examine the binding of rPoCXCL10 to bacteria components, lipopolysaccharide (LPS), peptidoglycan (PGN) and lipoteichoic acid (LTA) (all from Sigma, St Louis, MO, USA) were dissolved in coating buffer to 50 μg/ml and added to 96-well plates (100 μl/well). After incubation at 4 °C for overnight, rPoCXCL10 binding was performed with ELISA as above.

To examine the binding of peptide to bacteria, the C-terminal α-helix region of PoCXCL10 (5’-KVVKDRLIRIIKK-3’), named P13, was synthesized by ChinaPeptides Co., Ltd. (Shanghai, China). P13 binding to *V. harveyi* and *V. anguillarum* was determined as above with slight adjustments. Briefly, the plates were coated with bacteria and blocked as above, then fluorescein isothiocyanate (FITC) labeled-P13 at various concentrations (1.25 μM, 5 μM, and 20 μM) was added to the plates. The plates were incubated at room temperature for 2h and washed five times with PBST. The fluorescence intensity was detected with a multifunctional microplate reader (TECAN Infinite M200 PRO, Switzerland). The peptide P86P15 was used as a negative control peptide (NC) based on the previous report (47). To examine the effect of P13 on rPoCXCL10 binding to bacteria, *V. anguillarum* and *V. harveyi* were incubated with 60 μM P13 or NC or PBS for 2 h and washed as above. The bacteria were then incubated with 25 nM rPoCXCL10 or rSumo or PBS for 2 h. The bacteria-bound protein was detected by ELISA as described above.

### Effect of rPoCXCL10 on Bacterial Growth and Survival

To determine the effect of rPoCXCL10 on bacterial growth, *V. anguillarum, V. harveyi, E. tarda* and *S. iniae* were cultured to logarithmic phase. *V. anguillarum*, *V. harveyi*, and *E. tarda* were suspended in LB medium to 1×10^5^ CFU/ml in 96-well plates (100 μl/well). *S. iniae* was suspended in tryptic soy broth (TSB) medium (Hopebio, Qingdao, China) to 1×10^5^ CFU/ml in 96-well plates (100 μl/well). The bacterial suspension was mixed with or without (control) rPoCXCL10 or rSumo to a final concentration of 4 μM. The plates were incubated at 28°C for 12 h with shaking, and OD_600_ was measured at every hour to determine the bacterial growth. To determine the effect of rPoCXCL10 on bacterial survival, *V. anguillarum*, *V. harveyi*, *E. tarda* and *S. iniae* were suspended in PBS to 1×10^3^ CFU/ml and mixed with rPoCXCL10 or rSumo (4 µM) or PBS (control). The samples were then incubated at room temperature for 4 h (at which time point, the bacterial membrane damage could be detected). After incubation, the number of survived bacteria was determined by plate count. The bactericidal activity of rPoCXCL10M was similarly performed. To determine the bactericidal activity of P13, *V. anguillarum* and *V. harveyi* were incubated with or without (control) 20 µM P13 or NC for 4 h. The number of survived bacteria was determined as above.

### Electron Microscopy and Propidium Iodide Staining Assay

*V. anguillarum* was cultured to logarithmic phase and resuspended in PBS to 1×10^6^ CFU/ml. The bacteria were incubated with 4 µM rPoCXCL10 or PBS at room temperature for 4 h. After incubation, the bacteria were washed and resuspended in ultrapure water and observed with a transmission electron microscope (TEM) (Hitachi, HT7700, Japan) as described previously (48). For the PI staining assay, *V. anguillarum* was pretreated with rPoCXCL10 or PBS as above, and then stained with PI (2 µg/ml) for 10 min. The bacteria were observed with a fluorescence microscope (TiS/L100, Nikon, Tokyo, Japan).

### Chemotaxis Assay

Chemotaxis assay was carried out in 24-well Transwell plates (Corning Costar Co., Cambridge, MA, USA) with the pore size of 3 μm. rPoCXCL10 (0.25 μM, 1 μM, or 4 μM) or rSumo (4 μM) or PBS (control) in 600 μl L-15 medium (Sigma, St Louis, MO, USA) was placed at the lower chamber of Transwell. Flounder peripheral blood leukocytes (PBLs) were prepared as reported previously (49) and adjusted to 1×10^7^ cells/ml in L-15 medium. The PBLs (100 μl) were loaded onto the upper chamber of the above Transwell plates. The plates were incubated at 22°C for 2 h, and the cells migrated into the lower chamber was observed and counted with a microscope. The chemotactic index was presented as the fold increase in the number of migrated PBLs induced by recombinant protein to that induced by PBS. The chemotactic activity of rPoCXCL10M was similarly determined. To examine the effects of LPS and PGN on the chemotactic activity of rPoCXCL10, 1 μM rPoCXCL10 was pre-incubated with or without 50 μg/ml LPS or PGN for 2 h, and the chemotactic activity of rPoCXCL10 was determined as above.

### Binding of rPoCXCL10/M to PBLs

Binding of recombinant proteins to PBLs was detected by immunofluorescence microscopy as reported previously (44) with slight adjustments. Briefly, Japanese flounder PBLs were resuspended in L-15 medium to 1×10^7^ cells/ml, and the cells were added to cell culture dish (NEST, USA) for 4 h to allow the cells to settle. The cells were then blocked with 5% skim milk and incubated at 22°C for 1 h. After washing with PBS, 1 μM rPoCXCL10, 1 μM rPoCXCL10M, or PBS were added to the dish and incubated at 22°C for 2 h, followed by washing with PBS for three times. The cells were treated with mouse anti-Flag antibody (ABclonal, Wuhan, China) at 1/1,000 dilution for 1h and then treated with Alexa Fluor^®^ 488-conjugated goat anti-mouse IgG antibody (Abcam, UK) at 1/2,000 dilution for 1 h, followed by washing with PBS for three times. The cells were fixed with 4% paraformaldehyde (Biosharp, Shanghai, China) for 30 min and washed as above, then the cells were stained with 1, 1′-dioctadecyl-3, 3, 3, 3′-tetramethylindocarbocyanine perchlorate (DiI) (Invitrogen, Carlsbad, CA, USA) and 4’, 6-diamidino-2-phenylindole (DAPI) (Beyotime, Shanghai, China). The cells were then observed with a Zeiss LSM 710 confocal microscope (Carl Zeiss, Oberkochen, Germany).

### The Effect of rPoCXCL10 on PBLs Gene Expression

Flounder PBLs (1×10^6^ cells/ml) were treated with 1 μM rPoCXCL10, 1 μM rSumo, or PBS at 22°C for 3 h, 6 h, and 12 h. Total RNA was extracted at each time point and used for qRT-PCR as described above to analyze the expression of IL-6, TNF-α, CXCL8, IL-1β, and IL-10. The sequences of the primers used for qRT-PCR are listed in **Table S1**.

### The Effects of rPoCXCL10 and rPoCXCL10M on Bacterial Infection of PBLs

*E. tarda* was cultured to logarithmic phase and resuspended in 100 μg/ml FITC (YuanYe Bio-Technology, Shanghai, China), followed by incubation at room temperature for 3 h. The bacteria were collected and washed five times with PBS. The bacteria were resuspended in L-15 medium to 1×10^8^ CFU/ml and incubated with or without (control) 1 μM rPoCXCL10, rPoCXCL10M, or rSumo at room temperature for 2 h. Flounder PBLs were mixed with untreated *E. tarda* or *E. tarda* treated with rPoCXCL10, rPoCXCL10M, or rSumo at a multiplicity of infection (MOI) of 1:2. After incubation at 22°C for 2 h, the PBLs were centrifuged and washed with PBS. Trypan blue (0.125% in PBS) was added to the cells to quench the extracellular fluorescence. The cells were washed and suspended in PBS. The intracellular fluorescence signal of the bacteria was determined using a FACScan flow cytometer (BD Biosciences, USA).

### The Effects of rPoCXCL10 and rPoCXCL10M on Bacterial Infection *In Vivo*

*E. tarda* and *V. anguillarum* were prepared as above. Japanese flounder were infected with 100 µL *E. tarda* (2×10^6^ CFU/ml) or 100 µL *V. anguillarum* (1×10^7^ CFU/ml) *via* intraperitoneal injection in the presence of 5 μM rPoCXCL10, 5 μM rPoCXCL10M, 5 μM rSumo or PBS. Liver, spleen, and head kidney were collected aseptically at 12 and 24 hpi for *V. anguillarum* infection or 12 and 36 hpi for *E. tarda* infection. Bacterial recoveries from the tissues were determined by plate count as reported previously (50).

### Statistical Analysis

All experiments were performed three times. Statistical analyses were carried out using GraphPad Prism version 6.01 (GraphPad Software Inc., San Diego, CA, USA). Data were analyzed with student’s t-test. The results were considered statistically significant when the *p* < 0.05.

## Results

### Sequence Characteristics of PoCXCL10

**Figure 1:**
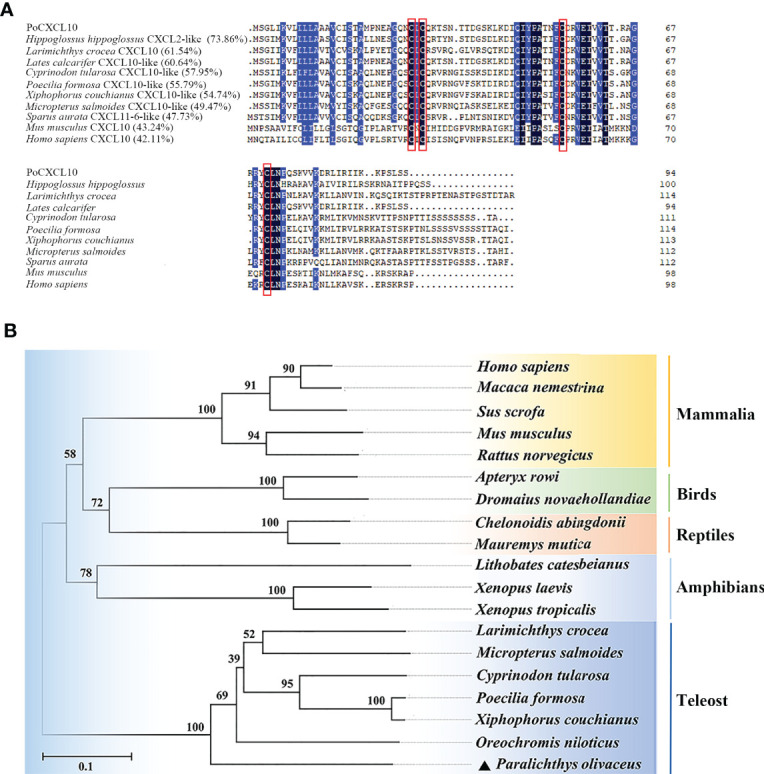
Sequence and phylogenetic analyses of PoCXCL10. **(A)** Sequence alignment of PoCXCL10 homologues. Dots denote gaps introduced for maximum matching. The conserved cysteine residues are boxed in red; the consensus residues are in black; the residues that are ≥75% identical among the aligned sequences are in blue. **(B)** Phylogenetic analysis of PoCXCL10. The phylogenetic tree was constructed using the neighbor-joining (NJ) method of the MEGA 6.0 package. The GeneBank accession numbers of the sequences are as follows: PoCXCL10, XP_019935349.1; *Hippoglossus hippoglossus*, XP_034453744.1; *Larimichthys crocea*, XP_019121063.2; *Lates calcarifer*, XP_018555122.1; *Cyprinodon tularosa*, XP_038155112.1; *Poecilia formosa*, XP_007548388.1; *Xiphophorus couchianus*, XP_027864958.1; *Micropterus salmoides*, XP_038555155.1; *Sparus aurata*, XP_030251220.1; *Mus musculus*, NP_067249.1; *Homo sapiens*, NP_001556.2; *Oreochromis niloticus*, XP_019222559.1; *Xenopus tropicalis*, XP_002940621.2; *Xenopus laevis*, XP_018098281.1; *Lithobates catesbeianus*, PIO23185.1; *Mauremys mutica*, XP_044875629.1; *Chelonoidis abingdonii*, XP_032623980.1; *Dromaius novaehollandiae*, XP_025959343.1; *Apteryx rowi*, XP_025945955.1; *Rattus norvegicus*, NP_620789.2; *Sus scrofa*, ABD18444.1; *Macaca nemestrina*, AAO52732.1.

PoCXCL10 is a 94-amino acid protein with an N-terminal signal peptide (residues 1 to 20) and four conserved cysteine residues (Cys-29, 31, 55 and 71) (**Figure 1A**). The predicted mature protein (without signal peptide) has a molecular weight of 8.3 kDa and a theoretical pI of 9.64. PoCXCL10 shares 42.11% and 43.24% overall sequence identities with *Homo sapiens* and *Mus musculus* CXCL10, respectively. Phylogenetic analysis showed that PoCXCL10 was grouped together with the putative fish CXCL10 homologues, which formed a distinct cluster separated from the clusters formed by the CXCL10 of amphibian, reptile, bird, and mammal (**Figure 1B**).

### The Expression of PoCXCL10 Is Regulated by Bacterial Pathogens

qRT-PCR showed that PoCXCL10 mRNA was present in nine tissues, i.e., liver, spleen, heart, brain, intestine, head kidney, muscle, blood, and gill, in the order of decreasing expression (**Figure S2A**). When flounder were infected with the bacterial pathogen *E. tarda*, PoCXCL10 expression was significantly up-regulated at 12, 24, and 48 hpi in spleen and head kidney, and at 6, 12, 24, and 48 hpi in liver (**Figure S2B**). Similarly, infections by the bacterial pathogens *V. anguillarum* and *V. harveyi* induced PoCXCL10 expression in flounder tissues in a time-dependent manner.

### rPoCXCL10 Binds Gram-Negative/Positive Bacteria and Affects Bacterial Growth and Survival by Inducing Membrane Damage

**Figure 2:**
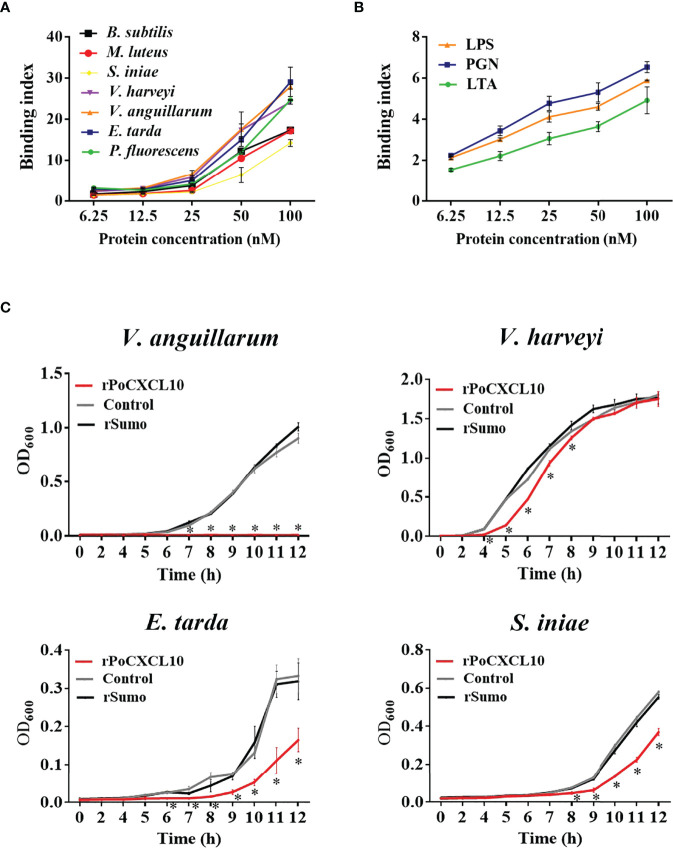
Interaction of rPoCXCL10 with bacterial components and its effect on bacterial growth. **(A)** *Bacillus subtilis*, *Micrococcus luteus*, *Streptococcus iniae*, *Vibrio anguillarum*, *Vibrio harveyi*, *Edwardsiella tarda*, and *Pseudomonas fluorescens* were incubated with or without (control) rPoCXCL10 at various concentrations (6.25 nM, 12.5 nM, 25 nM, 50 nM, or 100 nM). Bacteria-bound protein was detected by ELISA. **(B)** lipopolysaccharide (LPS), peptidoglycan (PGN) and lipoteichoic acid (LTA) were incubated with or without (control) rPoCXCL10 (6.25 nM, 12.5 nM, 25 nM, 50 nM, or 100 nM), the bound protein was determined by ELISA. **(C)** *V. anguillarum*, *V. harveyi*, *E. tarda*, and *S. iniae* were cultured in the presence or absence (control) of 4 μM rPoCXCL10 or 4 μM rSumo, and bacterial growth at different time points was determined by measuring absorbance at OD_600_. Values are the means of triplicate experiments and shown as means ± SD, *p < 0.05.

ELISA showed that rPoCXCL10 was able to bind both Gram-positive bacteria (*B. subtilis*, *M. luteus*, and *S. iniae*) and Gram-negative bacteria (*V. harveyi*, *V. anguillarum*, *E. tarda*, and *P. fluorescens*) in a dose-dependent manner (**Figure 2A**). rPoCXCL10 also bound apparently to pathogen-associated molecular patterns (PAMPs), i.e., LPS, PGN, and LTA (**Figure 2B**).

**Figure 3:**
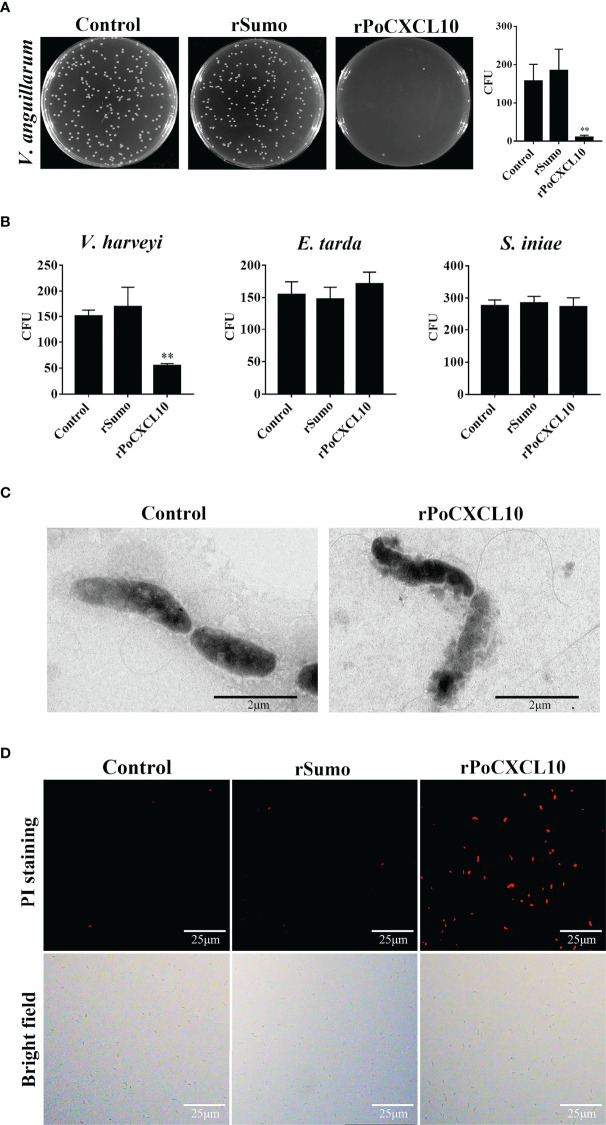
The bactericidal activity of rPoCXCL10. **(A)** *Vibrio anguillarum* was incubated with rPoCXCL10 or rSumo or PBS (control) for 4 h. The bacterial cells were plated on Luria-Bertani (LB) plates and incubated at 28°C for 12 h. The number of colony-forming unit (CFU) was counted and shown on the right. **(B)** *Vibrio harveyi*, *Edwardsiella tarda* and *Streptococcus iniae* were incubated with rPoCXCL10 or rSumo or PBS as above, and bacterial survival was determined as above. **(C)** *V. anguillarum* was incubated with rPoCXCL10 or PBS (control) for 4 h and then examined with transmission electron microscopy. **(D)** *V. anguillarum* were incubated with rPoCXCL10 or rSumo or PBS (control) for 4 h, and then stained with propidium iodide (PI). The cells were observed with a fluorescence microscope. Values are the means of triplicate experiments and shown as means ± SD. **p < 0.01.

For *V. anguillarum*, *E. tarda*, and *S. iniae*, their growths were markedly inhibited by rPoCXCL10; for *V. harveyi*, its growth was inhibited transiently by rPoCXCL10 at the early stage (4-8 h) (**Figure 2C**). rPoCXCL10 binding significantly reduced the viability of *V. anguillarum* and *V. harveyi*, but had no effect on the survival of *E. tarda* or *S. iniae* (**Figures 3A, B**; **Figure S3**). Microscopy showed that rPoCXCL10 treatment damaged the cellular structure and membrane of *V. anguillarum*, which enabled propidium iodide (PI) to pass into the cells (**Figures 3C, D**).

### A PoCXCL10-Derived Peptide Exhibits Bactericidal Activity

**Figure 4:**
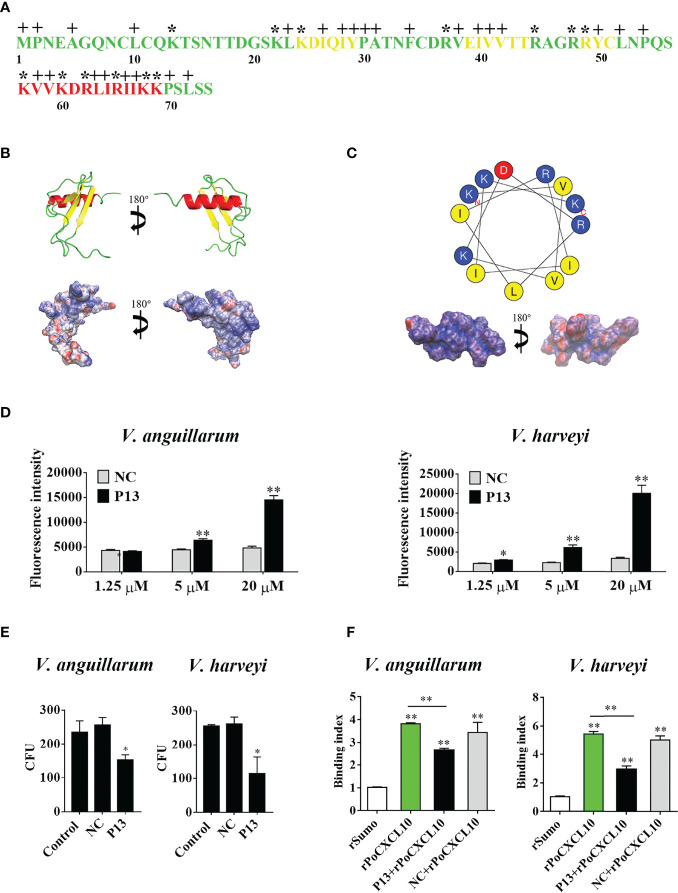
The structure and antibacterial activity of P13. **(A)** The primary structure of PoCXCL10. Sequences in green, yellow, and red stand for the extended coil, β-sheet and α-helix region, respectively. Positively charged residues and hydrophobic residues are marked by “*” and “+”, respectively. **(B)** The secondary structure (upper) and electrostatic surface potential (lower) of PoCXCL10. In the upper panel, green, yellow, and red stand for coil, β-sheet, and α-helix, respectively. In the lower panel, the positive- and negative-charged regions are in blue and red, respectively. **(C)** The helical wheel (upper) and solvent accessibility representation (lower) of P13. In the upper panel, the residues that are positively charged, negatively charged, and hydrophobic are in blue, red, and yellow, respectively. In the lower panel, the hydrophilic and hydrophobic regions are in blue and red, respectively. **(D)** *Vibrio anguillarum* and *Vibrio harveyi* were incubated with different concentrations of FITC-labeled P13 or the negative control peptide (NC), and positive binding was determined by measuring fluorescence intensity. **(E)** *V. anguillarum* and *V. harveyi* were incubated with P13, NC, or PBS (control) for 4 h. The bacteria were plated on LB plates and incubated at 28 °C for 12 h. The number of colony-forming unit (CFU) on the plates was counted and shown on the right. **(F)** *V. anguillarum* and *V. harveyi* were pre-incubated with or without P13 or NC, and then incubated with rPoCXCL10. Bacteria-bound protein was detected by ELISA. For panels **(D–F)**, values are the means of triplicate experiments and shown as means ± SD. *p < 0.05, **p < 0.01.

Structurally, PoCXCL10 is composed of an extended N-terminal coil, three antiparallel β-strands, and a C-terminal α-helix (**Figure 4A**). PoCXCL10 contains abundant positively charged amino acid residues, in particular at the C-terminal α-helix, resulting in an apparent cationic surface (**Figure 4B**). Based on this structural feature, the C-terminal α-helix domain (residues 57-69) of PoCXCL10 was synthesized as a peptide named P13, which has a pI of 11.17. In the helical wheel view of P13, the predominant polar (cationic) amino acids and the hydrophobic amino acids were distributed on two different sides (**Figure 4C**). Solvent accessibility representation also showed distinct hydrophobic and hydrophilic areas (**Figure 4C**), suggesting an amphipathic characteristic of P13 like that of the classical antimicrobial peptides. In line with this observation, P13 was able to bind *V. harveyi* and *V. anguillarum* in a dose-dependent manner and significantly reduced the viability of the bacteria (**Figures 4D, E**). Furthermore, pre-treatment of the bacteria with P13 significantly reduced the binding of rPoCXCL10 to the bacteria (**Figure 4F**).

### rPoCXCL10 Regulates the Migration and Gene Expression of Flounder Cells

**Figure 5:**
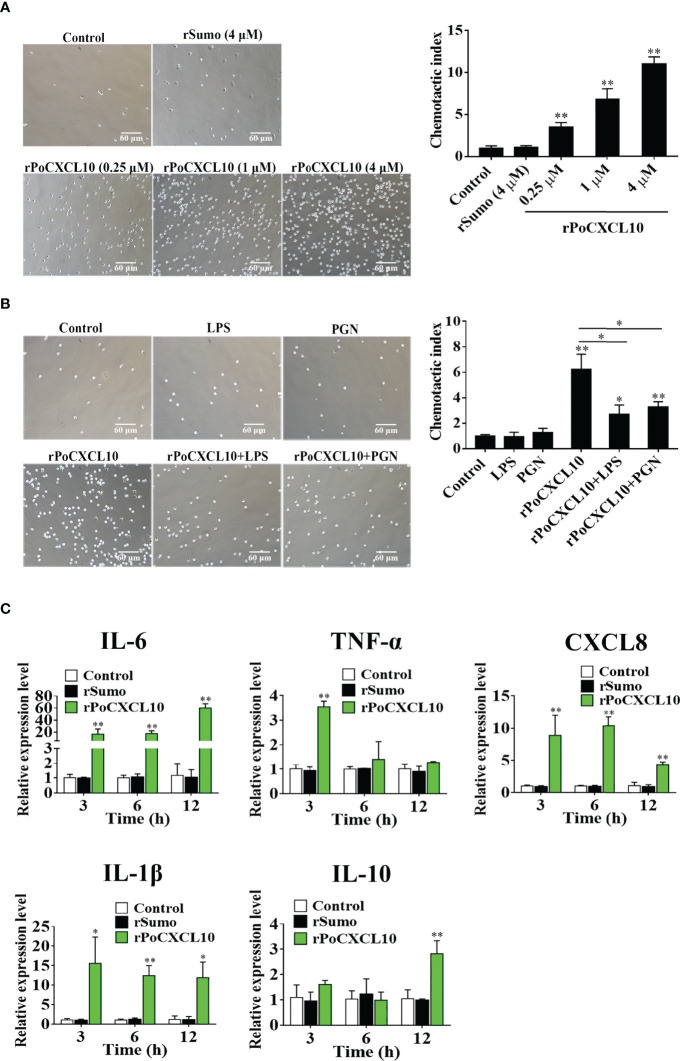
The effect of rPoCXCL10 on the migration and gene expression of flounder peripheral blood leukocytes (PBLs). **(A)** Flounder PBLs were treated with different concentrations of rPoCXCL10 or rSumo or PBS (control). Migration of the cells was determined by the Transwell migration assay. Bar size, 20 μm. Chemotactic index was presented as the ratio of the number of migrated cells induced by rPoCXCL10 or rSumo to that induced by PBS. **(B)** Flounder PBLs were incubated with or without (control) LPS, PGN, rPoCXCL10, or LPS/PGN-pretreated rPoCXCL10. Cell migration was then determined as above. The chemotactic index is shown in the graph. Bar size, 20 μm. **(C)** Flounder PBLs were treated with or without (control) rPoCXCL10 or rSumo for different hours, and the expression of inflammatory cytokines was determined by qRT-PCR. For all panels, values are the means of triplicate experiments and shown as means ± SD. *P < 0.05; **P < 0.01.

Transwell migration assay showed that rPoCXCL10 could induce the migration of flounder PBLs in a dose-dependent manner (**Figure 5A**). However, pre-treatment with LPS or PGN significantly reduced the chemotactic activity of rPoCXCL10 (**Figure 5B**). qRT-PCR showed that in rPoCXCL10-treated PBLs, the expressions of IL-6, CXCL8, and IL-1β were significantly up-regulated at 3, 6, and 12 h, and the expressions of TNF-α and IL-10 were significantly up-regulated at 3 and 12 h, respectively (**Figure 5C**).

### The C-Terminal α-helix of PoCXCL10 Is Required for Direct Bactericidal Activity but not for Chemotaxis or *In Vivo* Antimicrobial Activity

**Figure 6:**
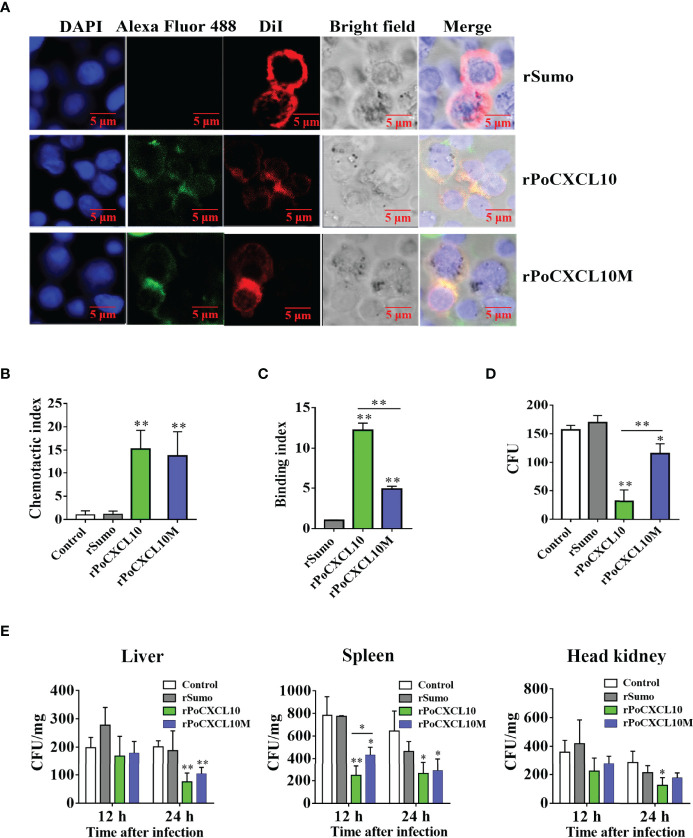
Comparison of the chemotactic and antimicrobial activities of rPoCXCL10 variants. **(A)** Binding of rPoCXCL10 and rPoCXCL10M to flounder peripheral blood leukocytes (PBLs). PBLs were incubated with rPoCXCL10, rPoCXCL10M, or the negative control protein rSumo for 2 h. The cell-bound protein was detected by Alexa Fluor^®^488-conjugated antibody. After staining with DiI and DAPI, the cells were observed with a confocal microscope. Bar size, 2 μm. **(B)** The chemotactic activities of rPoCXCL10 and rPoCXCL10M against PBLs were determined using Transwell migration assay. Chemotactic index was presented as the ratio of the number of migrated cells induced by rPoCXCL10 or rPoCXCL10M to that induced by PBS (control). **(C)** *Vibrio anguillarum* was incubated with rPoCXCL10, rPoCXCL10M, rSumo, or PBS. Bacteria-bound protein was detected by ELISA. **(D)** *V*. *anguillarum* was incubated with rPoCXCL10, rPoCXCL10M, rSumo, or PBS (control) for 4 h. The number of survived bacteria was determined by plate count. **(E)** Flounder were infected with *V. anguillarum* in the presence or absence (control) of rPoCXCL10, rPoCXCL10M, or rSumo. At 12 and 24 h post infection, bacterial numbers in the liver, spleen, and head kidney were determined by plate count. For panels **(B–E)**, values are the means of triplicate experiments and shown as means ± SD. *p < 0.05, **p < 0.01.

To examine the importance of the C-terminal α-helix to the immune functions of PoCXCL10, a mutant PoCXCL10 lacking this region was prepared as recombinant protein named rPoCXCL10M. Subsequent analysis showed that both rPoCXCL10 and rPoCXCL10M were able to bind to flounder PBLs and exhibited comparable chemotactic activities (**Figures 6A, B**). Fluorescence-activated cell sorting (FACS) analysis showed that both rPoCXCL10 and rPoCXCL10M significantly enhanced bacterial phagocytosis by PBLs (**Figure S4A**). However, compared to rPoCXCL10, rPoCXCL10M exhibited significantly reduced abilities to bind and kill bacteria (**Figures 6C, D**). When flounder were infected with *V. anguillarum* in the presence of rPoCXCL10 or rPoCXCL10M, the bacterial loads in liver (12 hpi) and spleen (12 hpi and 24 hpi) were significantly reduced compared to that in the control fish (**Figure 6E**). The reduction levels caused by rPoCXCL10M were largely comparable to that caused by rPoCXCL10 (**Figure 6E**). Similar results were obtained when the infection was performed with *E. tarda* (**Figure S4B**).

## Discussion

The chemokine superfamily has a large number of members belonging to different subfamilies, many of which have been demonstrated to play vital roles in innate and adaptive immunity (3, 4, 51–53). CXCL10 is a member of the CXC chemokine subfamily. To date, several CXCL10 from different fish species have been identified, and their mRNA expression levels before and after pathogen infection were determined (35–39). However, the biological activity of fish CXCL10 as a protein remains largely unclear. In this study, we characterized the function of a flounder CXC chemokine, PoCXCL10, in association with antimicrobial immunity. Structurally, PoCXCL10 possesses the four cysteine residues conserved in mammalian CXC, which form two intramolecular disulfide bonds that stabilize the molecule (54). Of the few reported fish CXCL10, they were shown to exhibit high levels of mRNA expression in immune relevant organs such as spleen, head kidney, and liver, and their expression levels could change significantly after bacterial challenge (35, 37, 38). Similarly, we found that PoCXCL10 expression was relatively high in liver and spleen, and was upregulated during the infection of *E. tarda*, *V. anguillarum*, and *V. harveyi*. These results indicate that PoCXCL10 is involved in pathogen-induced immune response. A previous study showed that in rainbow trout, CXCL10 expression was not induced by LPS (39). It is likely that LPS and live bacteria differ in some aspects of their immunoregulations on the expression of host genes such as CXCL10.

Some human chemokines, such as CXCL14 and CXCL9, kill bacteria directly (55, 56). In fish, rainbow trout CK11 and grass carp CXCL20a/b were reported to possess bactericidal activities (21–23). In our study, we found that rPoCXCL10 interacted directly with both Gram-negative and Gram-positive bacteria. These interactions occurred likely *via* PAMPs on the part of the bacteria, because rPoCXCL10 exhibited apparent binding to LPS, the central components of the outer membrane in Gram-negative bacteria, and PGN, the cell wall of Gram-positive bacteria. rPoCXCL10 binding inhibited the growth of *V. anguillarum*, *V. harveyi*, *E. tarda*, and *S. iniae*, which are severe pathogens to a wide range of fish species including flounder. rPoCXCL10 displayed apparent bactericidal activity against *V. anguillarum* and *V. harveyi*. These observations indicate that rPoCXCL10 can act as a bactericide or a bacteriostat, depending on the target bacteria. Microscopy revealed that the rPoCXCL10 treatment disrupted the membrane and cellular structure of the target bacteria, which was the direct cause of bacterial death.

In mammals, CXCL10 exhibits chemotactic activity towards various types of cells, including T cells, natural killer cells, and macrophages (57–59). In this study, we found that rPoCXCL10 could induce the migration of PBLs in a dose-dependent manner, indicating that rPoCXCL10 is an active chemoattractant. It is interesting that the presence of LPS or PGN significantly decreased the chemotactic activity of rPoCXCL10. It is possible that binding to LPS or PGN may affect the binding of rPoCXCL10 to its receptors on PBLs, or obstructing the effective signal transduction mediated by rPoCXCL10-receptor interaction. Mammalian CXCL10 is known to induce the production of inflammatory cytokines such as TNF-α, IL-1β, and CCL2 (29). Similarly, we found that rPoCXCL10 enhanced the expression of a variety of inflammatory cytokines in PBLs, indicating an immune regulatory function of rPoCXCL10.

It is well known that most antimicrobial peptides (AMPs) possess cationic charges and amphipathicity, which enable the AMPs to interact with the plasma membrane of the target bacteria (60). Structure modeling revealed that the C-terminal α-helix region of PoCXCL10 is amphipathic and highly positively charged. In agreement with this feature, a peptide, P13, derived from the α-helix was able to bind and kill *V. anguillarum* and *V. harveyi*. Hence, P13 probably retained the bactericidal activity of PoCXCL10. In the presence of P13, the rPoCXCL10-bacteria interaction was significantly hindered, implying that P13 competed with rPoCXCL10 for the same or overlapping binding sites in bacteria. These results suggest that the C-terminal α-helix is responsible, at least in part, for the interaction between PoCXCL10 and bacteria. In line with these results, deletion of the C-terminal α-helix region from PoCXCL10 significantly reduced the bacteria binding ability and the bactericidal activity of PoCXCL10, but did not affect the ability of PoCXCL10 to bind PBLs or induce chemotactic signaling, which was consistent with the previous observations that for mammalian chemokines, the N-terminal region was essential for receptor binding (61, 62). In the present study, *in vivo* infection showed that both rPoCXCL10 and rPoCXCL10M significantly reduced *V. anguillarum* and *E. tarda* dissemination in flounder tissues. Therefore, although the C-terminal α-helix of PoCXCL10 was essential to *in vitro* antibacterial activity, it was largely dispensable for *in vivo* antibacterial immunity under the experimental condition. It is likely that in the *in vitro* system, which contained no host cells, but only the bacteria and the chemokine, the antibacterial effect was solely dependent on the bactericidal activity of rPoCXCL10 or rPoCXCL10M, while *in vivo*, the antibacterial effect was mainly dependent on the ability of rPoCXCL10 or rPoCXCL10M to recruit and activate immune cells that eventually led to bacterial clearance.

In conclusion, our study demonstrated that PoCXCL10 was an active chemokine that regulated host cell immune response and promoted *in vivo* bacterial clearance. PoCXCL10 was also a bactericide and bacteriostatic that killed or inhibited the growth of fish pathogens. Importantly, we found that the C-terminal α-helix region of PoCXCL10 was crucial for bactericidal activity but not for chemotactic activity. These results add new insights into the function and mechanism of fish chemokines in antibacterial immunity.

## Data Availability Statement

The datasets presented in this study can be found in online repositories. The names of the repository/repositories and accession number(s) can be found in the article/**Supplementary Material**.

## Ethics Statement

The animal study was reviewed and approved by Ethics Committee of Institute of Oceanology, Chinese Academy of Sciences.

## Author Contributions

LS conceived the study; HL and YS conducted the experiments and analyzed the data; HL wrote the first draft of the manuscript; LS edited the manuscript. All authors contributed to the article and approved the submitted version.

## Funding

This work was supported by the grants from the National Key Research and Development Program of China (2018YFD0900500) and the Taishan Scholar Program of Shandong Province.

## Conflict of Interest

The authors declare that the research was conducted in the absence of any commercial or financial relationships that could be construed as a potential conflict of interest.

## Publisher’s Note

All claims expressed in this article are solely those of the authors and do not necessarily represent those of their affiliated organizations, or those of the publisher, the editors and the reviewers. Any product that may be evaluated in this article, or claim that may be made by its manufacturer, is not guaranteed or endorsed by the publisher.
