# Supplementary material for: A Teleost CXCL10 Is Both an Immunoregulator and an Antimicrobial
Source: Front Immunol. 2022 Jun 20;13:917697. doi: 10.3389/fimmu.2022.917697 (PMC9251016; doi:10.3389/fimmu.2022.917697)
Supplement: Supplementary file 1 [file DataSheet_1.docx]

**Supplemental data**

**Table S1.** List of primers used in this study.

| Primer | Sequences (5′→3′) ^a^ |
| --- | --- |
| PoCXCL10-F | GAACAGATTGGTGGTGGATCCATGATGCCTAACGAAGCCGGACA (BamH Ⅰ) |
| PoCXCL10-R | GTGGTGGTGGTGGTGCTCGAGTCAGCTGGACAGACTGGGTTTTTTGAT (Xho Ⅰ) |
| PoCXCL10M-F | GAACAGATTGGTGGTGGATCCATGATGCCTAACGAAGCCGGACA (BamH Ⅰ) |
| PoCXCL10M-R | GTGGTGGTGGTGGTGCTCGAGTCATGACTGGGGGTTCAGGCAGTAG (Xho Ⅰ) |
| RT-PoCXCL10-F | CCTAACGAAGCCGGACAGAA |
| RT-PoCXCL10-R | ACAGAAATTGGTTGCCGGGT |
| β-actin-F | AACCGCTGCCTCCTCCTCAT |
| β-actin-R | TCGGGACAACGGAACCTCTC |
| 18S rRNA-F | GGTCTGTGATGCCCTTAGATGTC |
| 18S rRNA-R | AGTGGGGTTCAGCGGGTTAC |
| α-tubulin-F | TGACATCACAAACGCCTGCTTC |
| α-tubulin-R | GCACCACATCTCCACGGTACAG |
| GAPDH-F | TCACTGCAACCCAGAAGACC |
| GAPDH-R | AGGGATGACCTTGCCAACAG |
| TNF-α-F | CCACAACACACTGAGGCAAA |
| TNF-α-R | TCCACACCAGCTTGTTTTCG |
| IL-6-F | TAACCGCTCACCACCAGAAA |
| IL-6-R | AGACAAGCCTCCTTGCTGAA |
| IL-1β-F | CAGCACATCAGAGCAAGACAACA |
| IL-1β-R | TGGTAGCACCGGGCATTCT |
| CXCL8-F | TGGCCATTCCTGATGGAACC |
| CXCL8-R | TCCACGCTTCCTATGTGACG |
| IL-10-F | GCCTCACGTGGAGTCCATAC |
| IL-10-R | CAGCTCGCCCATGGCTTTAT |

^a^Underlined nucleotides are restriction sites.

**Figure S1.** SDS-PAGE analysis of purified recombinant proteins. Purified rPoCXCL10, rPoCXCL10M, and rSumo were analyzed by SDS-PAGE and viewed after staining with Coomassie brilliant blue R-250. M, protein markers.

_
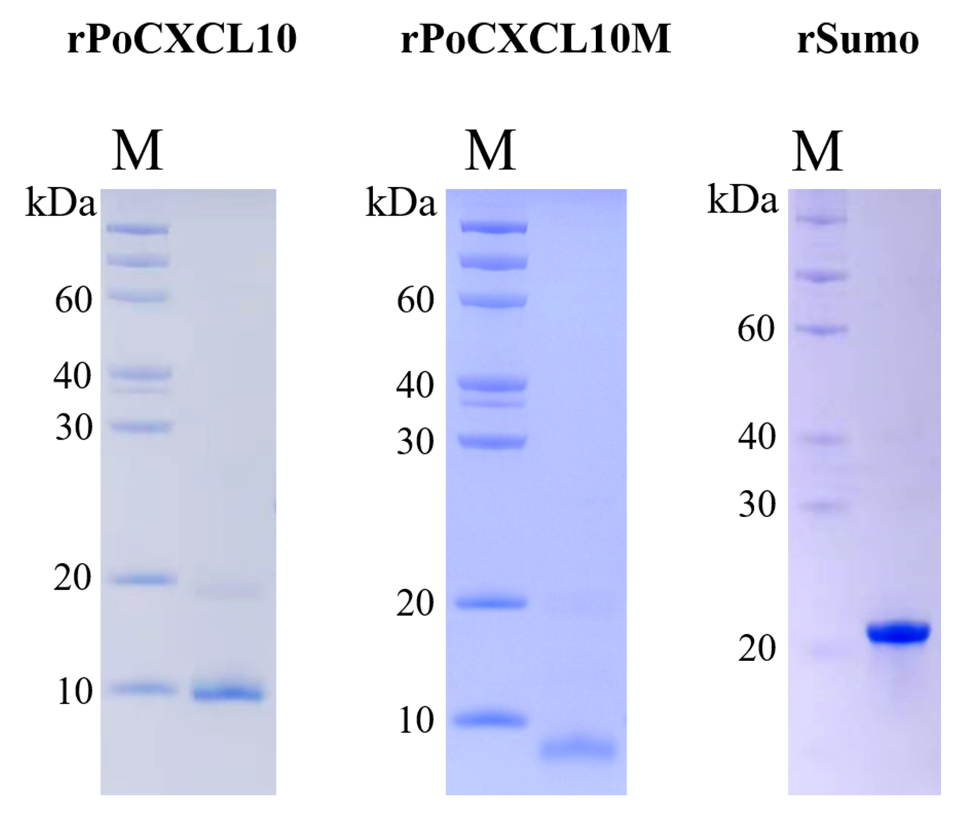
­­_

**Figure S2.** The expression of PoCXCL10 in flounder tissues. (A) PoCXCL10 mRNA expression in nine tissues of flounder was determined by qRT-PCR, with the expression level in the gill was set as 1. (B) Flounder were infected with or without (control) *Edwardsiella tarda*, *Vibrio anguillarum* or *Vibrio harveyi* for different hours, and the expression of PoCXCL10 in spleen, liver and head kidney was determined by qRT-PCR. Values are the means of triplicate experiments and shown as means ± SD. **p* < 0.05, ***p* < 0.01.

­
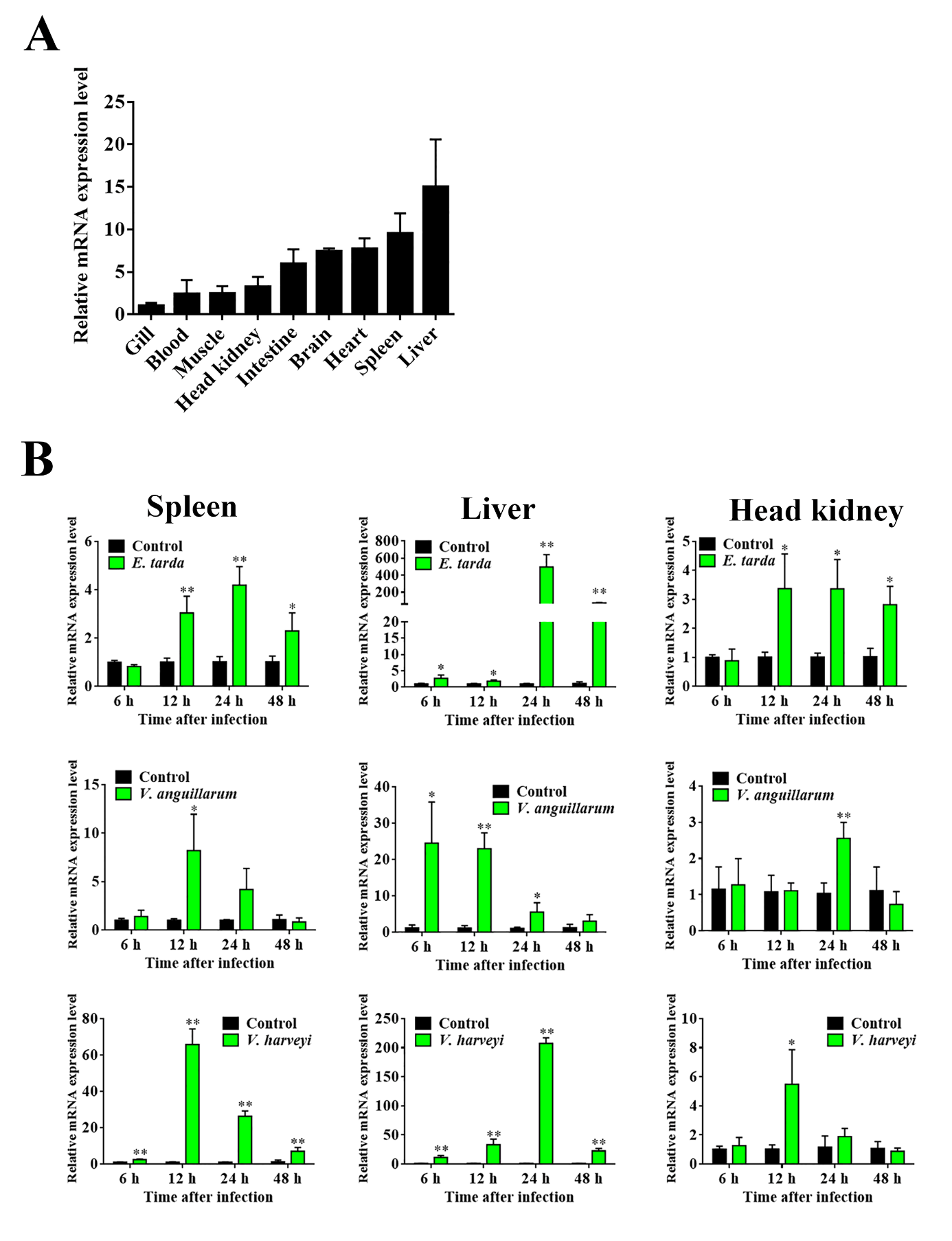


**Figure S3.** The bactericidal activity of rPoCXCL10 against *Vibrio harveyi*. *V. harveyi* was incubated with rPoCXCL10 or rSumo or PBS (control) for 4 h. The bacterial cells were plated on Luria-Bertani agar plates. The plates were incubated at 28 ℃ for 12 h and observed for bacterial growth.

**
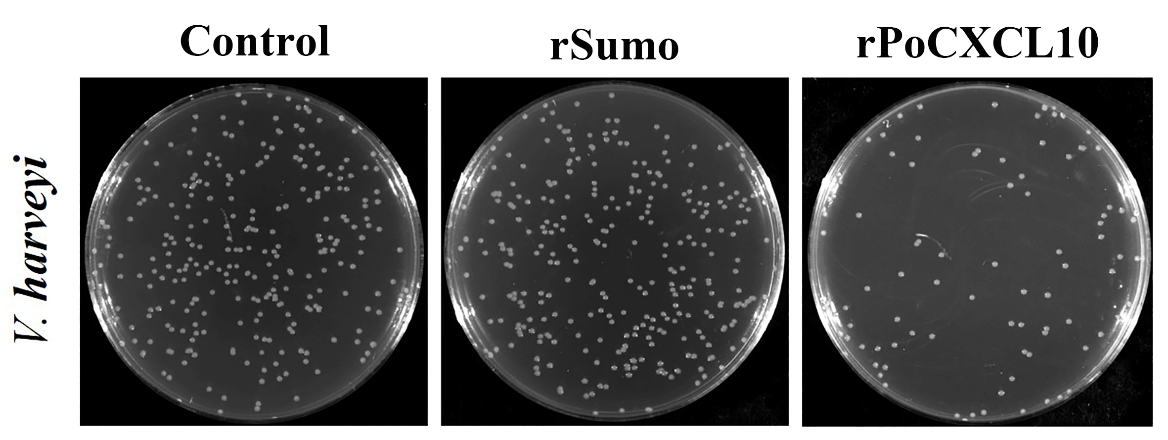
**

**Figure S4.** Effects of rPoCXCL10 and rPoCXCL10M on leukocyte phagocytosis and *in vivo* bacterial clearance. (A) Flounder peripheral blood leukocytes (PBLs) were incubated with FITC-labeled *E. tarda* pre-treated with or without (control) rPoCXCL10, rPoCXCL10M, or the control protein rSumo. Bacterial internalization was determined by flow cytometry. The internalization rates are shown in the graph on the right. (B) Flounder were infected with *E. tarda* in the presence or absence (control) of rPoCXCL10, rPoCXCL10M, or the control protein rSumo for 12 and 36 h, bacterial numbers in the liver, spleen, and head kidney were determined by plate count. Values are the means of triplicate experiments and shown as means ± SD. ***p* < 0.01.

_
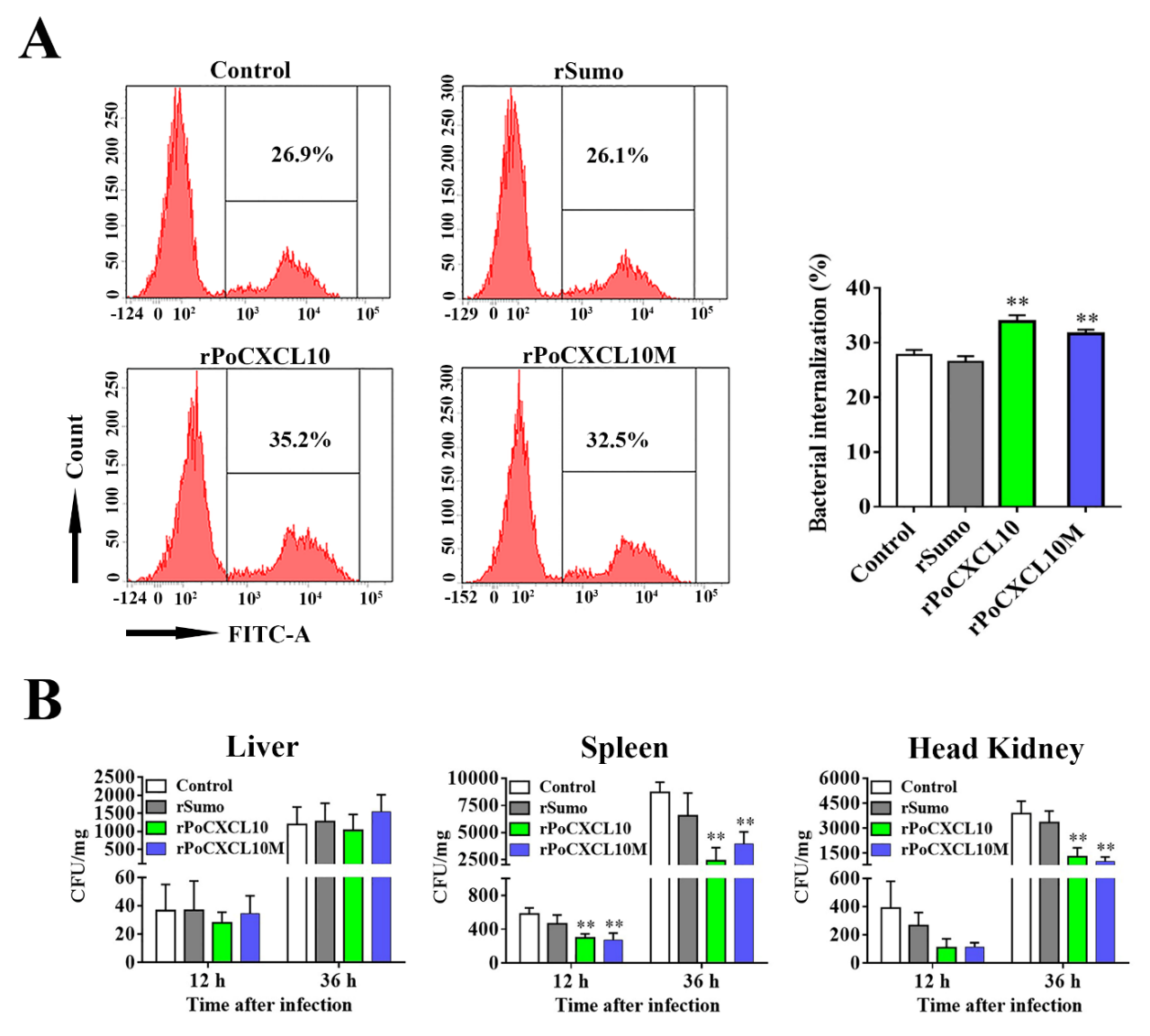
_
